# Patient experiences and decision-making priorities in early esophageal cancer: a qualitative interview study

**DOI:** 10.1093/dote/doag096

**Published:** 2026-09-11

**Authors:** Kirsty Cole, Pradeep Bhandari, Michael White, Tim Underwood, Sheraz Markar, Philip Pucher, the Association of Upper GI Surgery GB&I (AUGIS)

**Affiliations:** Department of General Surgery, Portsmouth University Hospitals NHS Trust, Portsmouth, UK; Division of Medicine, Pharmacy and Biomedical sciences, University of Portsmouth, Portsmouth, UK; Department of Gastroenterology, Portsmouth University Hospitals NHS Trust, Portsmouth, UK; Department of Gastroenterology, Portsmouth University Hospitals NHS Trust, Portsmouth, UK; School of Cancer Sciences, University of Southampton, Southampton, UK; Nuffield Department of Surgical Sciences, University of Oxford, Oxford, UK; Department of General Surgery, Portsmouth University Hospitals NHS Trust, Portsmouth, UK; Division of Medicine, Pharmacy and Biomedical sciences, University of Portsmouth, Portsmouth, UK; Department of General Surgery, Imperial College London, London, UK

**Keywords:** advanced endoscopy, early esophageal cancer, endoscopic resection, esophageal cancer surgery, esophagogastric surgery, quality of life

## Abstract

Endoscopic resection (ER) may offer organ preserving treatment in early (cT1N0) esophago-gastric (OG) cancer. Resectional surgery after ER may be recommended if pathological factors suggesting a risk of occult nodal metastasis are identified. Decision-making in this setting is complex, as patients must weigh the unknown risk of cancer progression with surveillance, against the more tangible and measurable impact of major surgery. Understanding patient priorities and values in making this decision is essential to support informed, patient-centered care. A semi-structured interview study was conducted. Patients who had previously undergone ER for early OG cancer were included. Patients were asked to consider a scenario in which they might be recommended either resectional surgery or endoscopic surveillance, and respond based on their own experiences and background. Interviews explored experiences of receiving the diagnosis, decision-making priorities, and joint decision-making preferences. Treatment-related anxiety was assessed using questions based on the validated cancer worry score. All interviews were audio-recorded, transcribed verbatim, and analyzed using thematic content analysis until data saturation was achieved. Thematic saturation was achieved after 17 interviews. Long-term quality of life and independence emerged as the most important factors influencing decision-making. Overall survival was highly valued, and the fear of operative mortality generally low. Cancer-related anxiety was common, particularly among patients who had recently undergone ER. Participants preferred to be involved and included in the decisions but generally wanted their clinicians to guide choices, and trusted their recommendations. Statistical risk information was often considered overwhelming or less useful for patients. The intensity of post-ER surveillance was a challenge for participants, with mixed impact. While many patients accepted this to avoid surgery, for others cancer-related anxiety or the burden of surveillance was reason to prefer surgical treatment. To conclude, decision-making after ER for early OG cancer reflects a complex interplay of physical, psychological, and logistical priorities, which may fit poorly into current guidelines for early OG cancer therapy. Patient-centered counseling must consider long-term quality of life, provide flexible communication, and incorporate balanced multidisciplinary input to facilitate informed and individualized decisions.

## INTRODUCTION

Endoscopic resection (ER) in early (cT1N0) esophago-gastric (OG) cancer may offer organ preserving therapy that avoids the morbidity and impact on quality of life associated with major surgery. Current guidelines recommend subsequent surgery in the case of histopathologically high-risk features such as deep submucosal invasion or lymphovascular invasion, due to the increased risk of occult lymph node metastases (LNM).^1–3^

Recent studies have reported substantial variability in rates of LNM in clinically staged T1N0 disease.^4^^,^^5^ These studies suggest that while the incidence of LNM may be higher than previously estimated, the majority of patients who undergo surgical resection for early OG cancer are ultimately found to have no nodal involvement. Therefore, some have suggested that even in high risk groups, endoscopic and radiological surveillance may be appropriate.^6^ Conversely, others have reported data showing a significant risk of nodal disease even in ‘low risk’ pathology.^4^^,^^5^

This dilemma highlights the difficult decision-making facing clinicians and patients choosing between treatment strategies; to balance the potential risk of occult disease, with the more tangible risks of major surgery. Previous studies in locally advanced cancer patients have reported that some patients are willing to accept a reduction in overall survival to avoid surgery.^7^^,^^8^ Such studies highlight the need to consider factors beyond cancer-related survival alone in joint decision-making. Psychological factors, financial concerns, and family wishes may play an equally important role in treatment decision-making. While patients managed with ER alone may avoid the potential negative impact of surgery, some studies have suggested that cancer-related anxiety in this setting may be comparable to, or even exceed that of patients who have undergone surgery for more advanced disease.^9–11^ Thus, patients’ decision-making is shaped not only by physical outcomes such as survival and morbidity but also by emotional responses to perceived risk and uncertainty. Such considerations may be exacerbated in early OG cancer, and will differ to groups previously studied, given the uncertainty of occult cancer risk in this setting.

To better understand these complex trade-offs, this study used semi-structured interviews to explore patient priorities when considering surgery following ER for T1 OG cancer. By examining how patients interpret risks, balance quality of life against the potential for disease recurrence, and weigh psychological factors such as anxiety, we aimed to identify the aspects of treatment decision-making that matter most to patients.

## METHODS

### Design and recruitment

A semi-structured interview was conducted following approval by a research ethics committee (HRA REC 25/NS/0074). The COREQ checklist was used in the design, analysis, and interpretation of the study.^12^ In order to explore priorities in the context of decision-making following ER, patients who had previously undergone ER for OG cancer, but had not proceeded to surgical resection, were recruited from across the South of England. Patients who had already undergone esophagectomy were not included, as prior experience of surgery may influence stated preferences and introduce retrospective bias when considering hypothetical treatment choices. To be eligible, participants needed to be at least 18 years of age and to be able to communicate in the English language. All participants gave informed consent to take part in the study. Potential participants were identified from an electronic database and approached directly. Semi-structured interviews were audio-recorded and transcribed.

### Interview structure

An interview script was designed, piloted, and finalized by senior clinicians with expertise in qualitative research and OG cancer management. The interview script was broken down into several domains: Patient background/demographics, receiving the diagnosis, priorities in decision-making, joint-decision making, cancer anxiety, and reflection. A combination of open and closed questions was used. Before the interviews began, a list of patient priorities when deciding between surveillance and surgery was devised based on clinical expertise and the existing literature to supplement patients’ open-ended responses.^7^^,^^8^^,^^13^ The interview script was piloted on the first three patients, and minor amendments to wording and question order were made. As these amendments did not alter the content of the interviews, the pilot patients were included in the final analysis.

Patients were asked about demographic details, performance status, and living arrangements. Patient postcodes were used to calculate the index of multiple deprivation (IMD) from publicly available data.

As part of exploring the joint decision-making pathway, patients were first asked about their experiences in receiving their original diagnosis. Patients were asked to consider what personal priorities might influence their decision-making, if the nature of their disease were to result in a choice of treatment between active endoscopic surveillance or major surgery. Participants were then asked about their tolerance to having risks/complications explained to them before a procedure, and about how involved they would like to be in decision making.

To understand the potential impact of anxiety and different personalities on interview responses, questions based on the validated Cancer Worry Scale (CWS)^14^ were included to establish the level of anxiety participants were experiencing whilst under surveillance for an endoscopically resected T1 OG cancer. The CWS is a validated questionnaire which asks patients to describe whether, or how frequently, they worry about topics across seven domains of cancer care. Each response is assigned a score from 0 to 3 and collated for a total score of 0 (least worry) to 21 (most worry).

### Analysis

All interviews were audio-recorded and transcribed verbatim. Each transcript was numerically coded and anonymized before undergoing thematic analysis using a conventional qualitative content analysis approach.^15^ Transcripts were reviewed in detail and coded according to recurring concepts and ideas, which were subsequently grouped into broader themes through discussion within the research team. Coding was primarily undertaken by the lead researcher (KC). 6/17 (35.3%) of the transcripts were also analyzed by an experienced qualitative researcher (PHP), to ensure calibration and congruity in coding. Any discrepancies were resolved through discussion. A sample size of 20 patients, or when thematic saturation was reached, was planned. Saturation was considered achieved when no new themes emerged in three consecutive interviews.

Descriptive analysis, including illustrative anonymized quotes, was used. Two-tailed Pearson correlation coefficients and Mann–Whitney U tests were used to explore associations between CWS scores and patient demographics, as well as preferences expressed during the interviews. For the latter, the effect size of any significant results (*P* < 0.05) were assessed by calculating the rank-biseral correlation (*r*).^16^ Effect sizes were interpreted according to Cohen’s criteria, whereby *r* > 0.1 and <0.3 indicates a small effect, *r* > 0.3 and <0.5 a medium effect, and *r* > 0.5 a large effect.^16^

## RESULTS

### Participants

Interviews were conducted in August and September 2025. All patients had previously undergone ER for OG cancer. Two consented participants withdrew prior to their interview due to unrelated medical problems. A pragmatic interim analysis was conducted after 17 interviews, at which point data saturation had been reached, so recruitment was stopped.

In total, 17 patients from eight different hospitals were interviewed, covering a broad geographic and socioeconomic catchment area. Fourteen out of 17 (82.4%) participants were male and the mean age at the time of ER was 71.6 years. IMD for participants ranged from 2 to 9 with a median of 7 (1 = most deprived, 10 = least deprived).

The period between endoscopic resection and interview ranged from 2 to 43 months, median 11 months (interquartile range [IQR] 7–27).

### Experiences with receiving the diagnosis

Ten out of 17 participants (58.8%) were content with the way in which their diagnosis was given to them. Primary complaints among the remaining seven participants included receiving the diagnosis over the telephone, not having a relative present for the conversation, and the use of medical jargon, which hindered understanding. Additionally, four participants felt insufficient time was allocated to the conversation, and two reported receiving their diagnosis immediately after endoscopy while still recovering from sedation. It was evident that some participants were unable to fully process the information, largely due to the shock of the diagnosis.

007: ‘It was just rather sudden. I think it would have been better if it had been a day after or if I could speak to someone a bit later and a bit more in depth because I’ve just had the endoscopy, you know it was all a bit sudden’.

Eleven out of 17 participants (64.7%) reported a good understanding of their diagnosis. However, one participant reported being unaware that they had ever been given a ‘cancer’ diagnosis (despite a history of ER for early OG cancer), and several asked questions about their condition during the interview, highlighting notable variability in patient comprehension and the potential for gaps in communication during the diagnostic process.

009; ‘Whether I just missed something somewhere I don’t know, but I didn’t realise how serious my condition was. It was a little bit of a shock when they did that, I suddenly thought things are more serious than I thought’

008; ‘When people ask me about the [endoscopic resection] I had, I vaguely point to the middle of my chest…which I think I where the surgery was, but I’m not exactly sure. And so in that sense, I am lacking particular details of knowledge’

A strong theme among participants was the desire for clear and honest information, with 14 out of 17 individuals (82.4%) emphasizing the importance of transparent communication when discussing treatment options. Participants consistently expressed a preference for direct, truthful explanations without unnecessary reassurance or minimization of potential outcomes.

004; ‘Just tell me the truth to be fair, give me the whole outcome, not just flower over it. I'm not the sort of person that's going to wilt’.

### Priorities in treatment decision-making

**Fig. 1:**
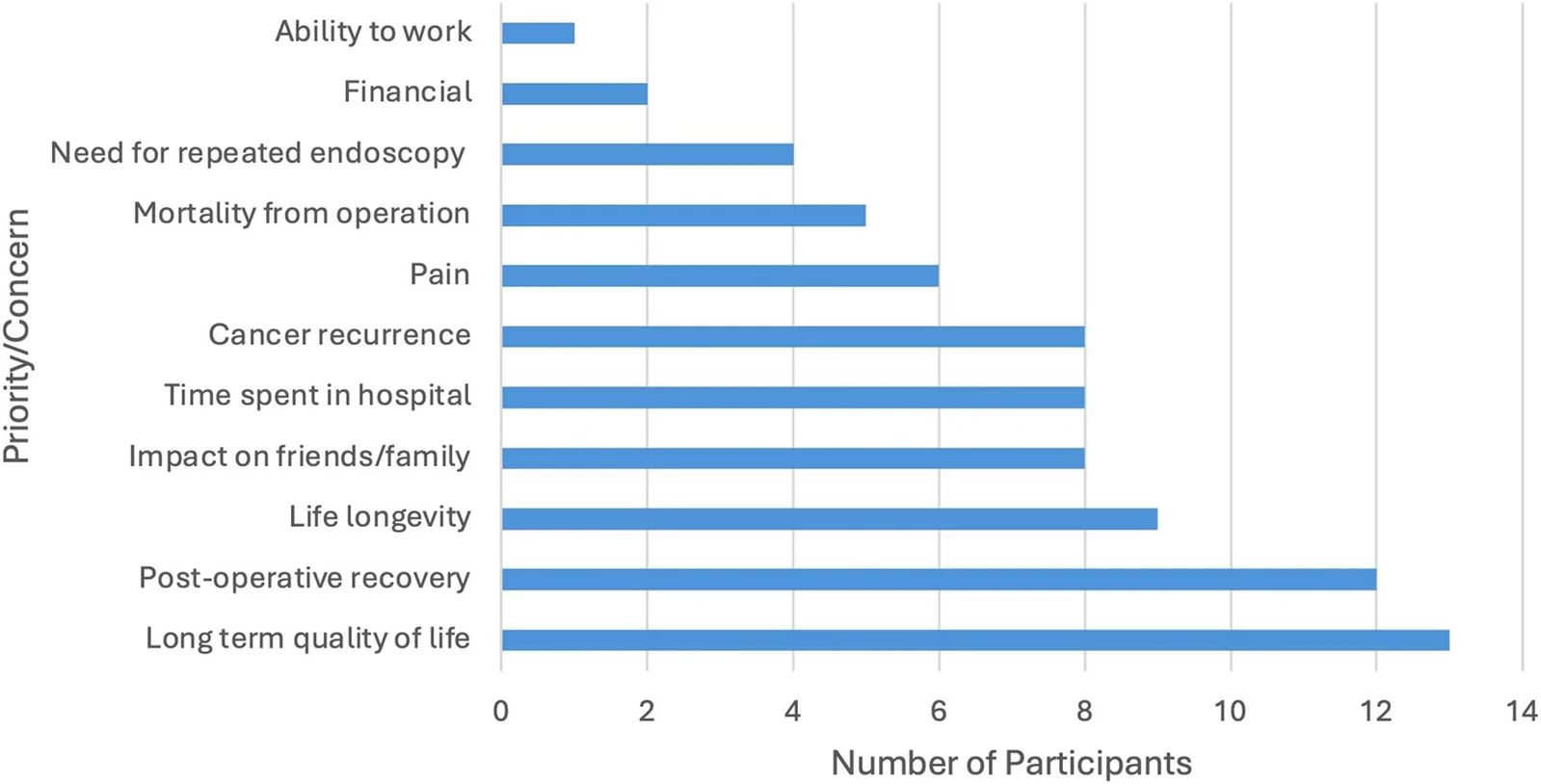
Patient priorities when considering major surgery versus active surveillance for an endoscopically resected early esophageal cancer.

Patient priorities influencing decision-making were grouped into 11 topics/themes (Fig. 1). Thirteen out of 17 (76.5%) highlighted that the possible impact on long-term quality of life would be a primary concern when deciding between surgery and surveillance. Many expressed fear of losing independence due to the effects of surgery on physical or mental function. Twelve out of 17 (70.6%) participants also highlighted anxiety regarding post-operative recovery in the form of immediate complications and short-term functional impact.

004: ‘It depends on my quality of life afterwards. I don't want to end up sitting at home doing nothing apart from watching the telly because that's not life’.

005: ‘How will I be affected when I come round and things like that. Because the last thing I really want is to get Alzheimers or dementia and I have a fear of that’.

Other identified concerns included the length of hospital stay (8/17, 47.1%), impact on family and friends (8/17, 47.1%), expected longevity with or without surgery (8/17, 47.1%), and risk of cancer recurrence (8/17, 47.1%). The latter group of participants indicated that surgery was preferable in some cases, as it was perceived to offer greater certainty regarding cancer clearance through removal of the tumor and surrounding tissue, with a consequent reduction in nodal recurrence risk.

019: ‘Well, basically how long would I have left… and then also quality of life in the few years afterwards. I’d want to know it was gone and wasn’t coming back. And the support of my family would be a big thing’.

005: ‘Well I’d like to know what my prospects are and, you know, how many years I’d have after the bad operation if I needed it’

Five out of 17 (29.4%) participants expressed concern over a quoted 2% surgical mortality risk, though the remaining participants considered it ‘low’. Twelve out of 17 (70.6%) participants traveled from other geographical regions for appointments; of these four out of 12 (33%) participants considered the travel burden of repeated surveillance investigations after ER to be a factor that would influence their preferred treatment pathway.

### Shared decision making preferences

When discussing joint decision making, 13 out of 17 (76.5%) participants expressed a desire to be involved in the process; however, this was often accompanied by a preference for clinician-led decision making. Participants commonly reported limited interest in receiving detailed information about the full range of risks associated with different treatment options. These accounts suggested that while participants value being included in conversations about their care, and want to understand it, they generally preferred that clinicians take the lead in making the final decisions and recommending the best course of action.

016; ‘I do like to be I like to be involved, but at the same time taking advice from whoever is giving it to me. I don't know anything about medical things, they do’.

015; ‘Well, I just want to be told what the options are, and that's it. That's all I need. I always leave it to the professional anyway’

005; ‘Well, I think whilst I’m in sound mind and body, I think it’s up to me to decide’.

When asked specifically if numbers or statistics (regarding risk of negative outcome or cancer recurrence, for example) would be of use to them in the decision-making process, the majority (10/17, 58.8%) said no, stating they find them ‘overwhelming’ or ‘daunting’, or that they ‘don’t always make sense’. Several of these participants (4/10, 40%) did however, acknowledge that such data may be useful for clinicians, even if they themselves found it difficult to interpret.

### Cancer related anxiety

**Fig. 2:**
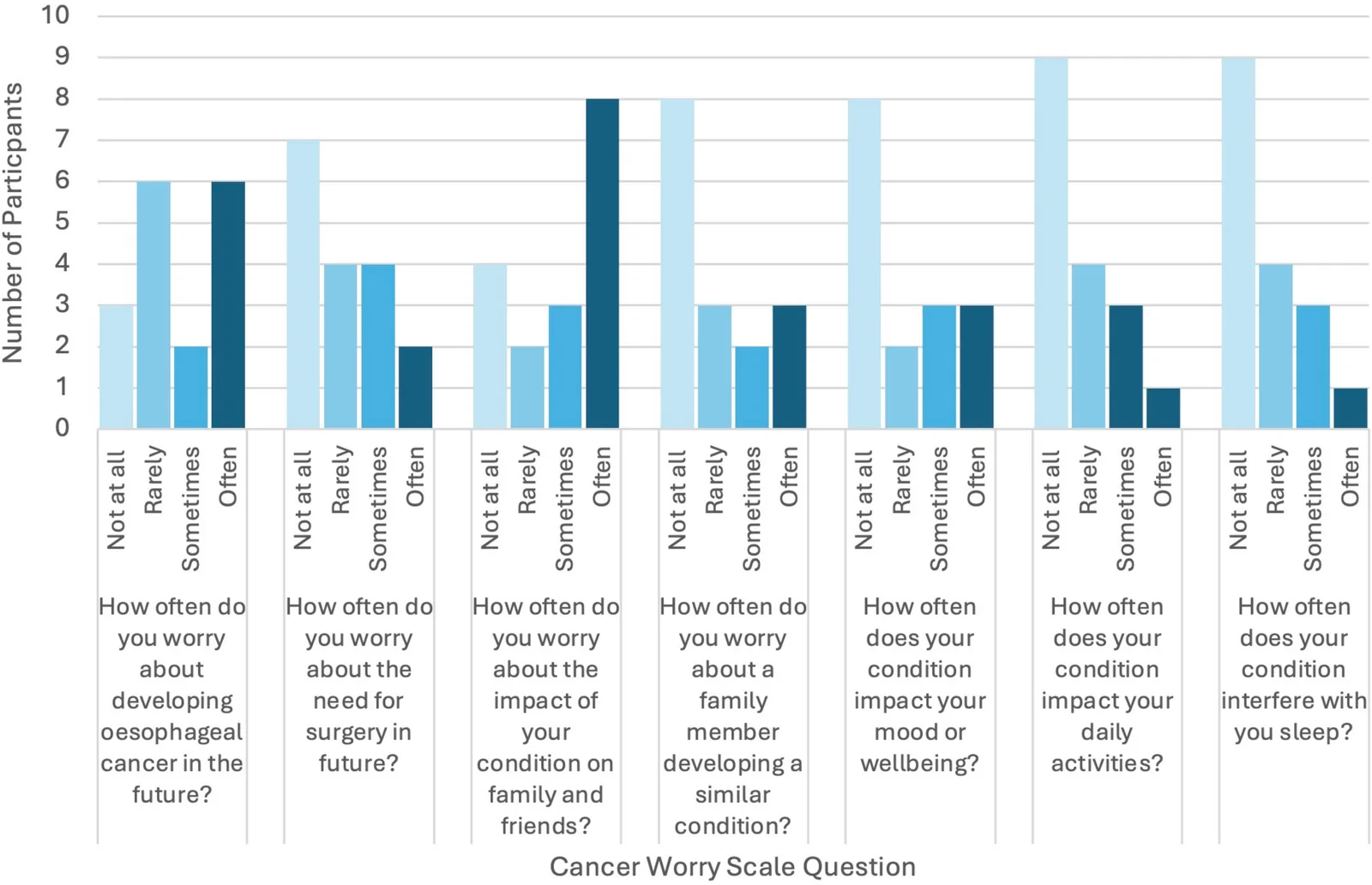
Cancer Anxiety Score in patients under surveillance for endoscopically resected T1 OG cancer.

Individual cancer worry scores ranged from 0 to 17 (median 10, IQR 3–11) (Table 1, Fig. 2). Most patients expressed anxiety over developing cancer again in the future (14/17, 82.4%), and about the possible impact of their condition on family and friends (13/17, 76.5%). Most patients answered ‘Not at all’ or ‘Rarely’ when asked about impact on mood (10/17, 58.8%), ability to carry out daily activities (13/17, 76.5%) and sleep (13/17, 76.5%).

**Table 1:** Patient demographics and characteristics of the 17 interview participants

**ID**	**Age**	**Gender**	**PS**	**IMD**	**Time from ER to interview (months)**	**CWS** ^*^
01	62	M	1	7	7	12
02	76	F	1	9	16	11
03	42	M	0	5	7	15
04	73	F	0	^†^	6	11
05	78	M	2	^†^	4	10
06	63	M	2	6	11	17
07	76	M	1	9	10	10
08	72	M	0	4	2	4
09	81	M	0	7	33	9
10	63	F	0	6	19	2
12	73	M	0	7	27	4
13	62	M	0	8	43	0
14	79	M	0	8	23	10
15	74	M	1	5	28	1
16	82	F	0	2	9	11
17	78	M	0	7	33	1
19	70	M	0	9	9	13

PS, performance status; IMD, index of multiple deprivation; CWS, Cancer Worry Score.

^*^Minimum = 0, maximum = 21.

^†^Not available as referred from outside England.

**Fig. 3:**
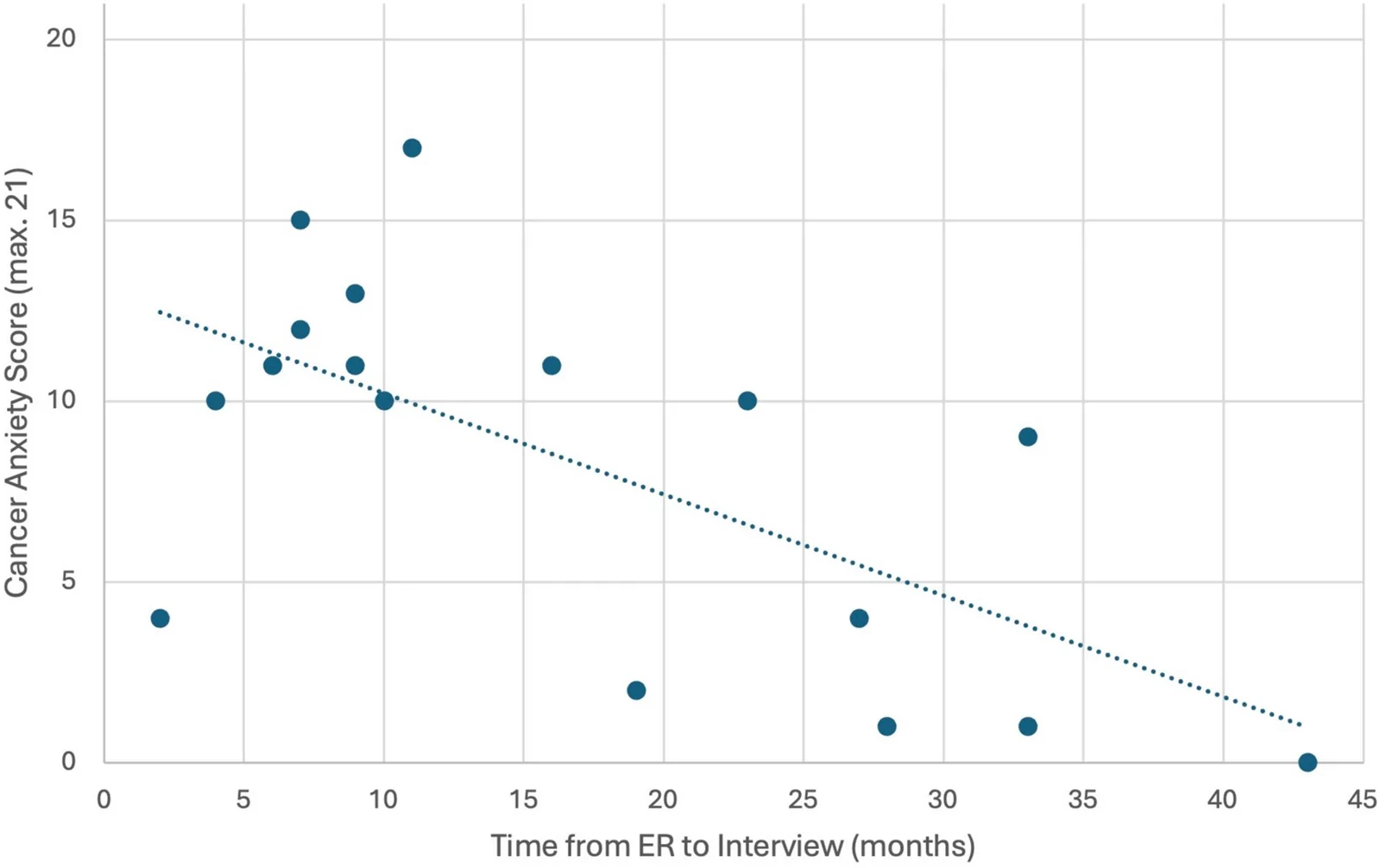
Correlation between cancer anxiety score and time in months from ER to interview (*P* = 0.005, effect size *r*^2^ = 0.422) (ER = Endoscopic resection).

There was no statistical correlation between the CWS and patients age, performance status, or socioeconomic background (*P* = 0.424, 0.156, and 0.854, respectively). There was, however, a statistically significant correlation between the CWS and the time between ER and interview (*P* = 0.005, *r*^2^ = 0.422) (Fig. 3), suggesting a moderate negative correlation between CWS and time from diagnosis.

Correlations between CWS and patient priorities are demonstrated in Table 2. There was a significant correlation between CWS, and increased anxiety over repeated endoscopies (*P* = 0.006) and the possible financial implications of surgery (*P* = 0.015). Both of these relationships suggested a large effect size (*r*^2^ = 0.635 and *r*^2^ = 0.546, respectively).

**Table 2:** Correlations between CWS patient priorities demonstrated by p values and effect size (*r*)

**Priority**	**Median CWS** ^*^ **of participants without this concern (IQR)**	**Median CWS** ^*^ **of participants with this concern (IQR)**	***P*-value**	**Effect size (*r²*)**
Ability to work	10 (2.5–11.0)	15 (15.0–15.0)	0.235	-
Financial	10 (1.0–11.0)	16 (15.0–16.0)	**0.015**	**0.546**
Need for repeat endoscopy appointments	9 (1.5–10.50)	13 (11.3–16.5)	**0.006**	**0.635**
Mortality from operation	10 (4.0–12.7)	11.5 (1.0–11.0)	0.721	-
Cancer recurrence	9 (3.0–10.0)	11.5 (3.5–14.5)	0.093	-
Time spent in hospital	10 (4.0–14.0)	9.5 (1.3–11.0)	0.423	-
Impact on friends/family	4 (1.0–12.0)	11 (9.3–11.7)	0.200	-
Life longevity	11 (7.0–13.50)	6.5 (1.0–10.7)	0.093	-
Post-operative recovery	10 (2.5–14.0)	10 (2.5–11.0)	0.799	-
Long-term quality of life	6 (1.3–10.0)	11 (4.0–12.5)	0.202	-

IQR, Interquartile range. ^*^Minimum=0, maximum=21.

Bold numbers represent statistical significance.

## DISCUSSION

This study provides an in-depth exploration of patient priorities when considering surgical resection versus organ-preserving treatment (ER and surveillance) for early OG cancer. These findings highlight the complex interplay between oncological, psychological, and lifestyle factors that influence patient decision-making in this context. Impact on long-term quality of life emerged as the most prominent concern among participants, for some outweighing considerations for overall survival. These findings highlight the importance of discussing not only oncological outcomes, but also the likely long-term functional consequences of each treatment option. Fears related to loss of independence, physical function, and mental capacity further shaped patient preferences. While immediate surgical risks and short-term functional consequences were also acknowledged, they were generally secondary to long-term considerations. Additional concerns, including potential need for hospital stay, family impact, intensity of surveillance, and expected longevity, further illustrate the multifactorial nature of shared decision-making in this context.

Previous interview studies have focused predominantly on patients following esophagectomy, retrospectively exploring experiences with diagnosis, treatment, and recovery.^17^^,^^18^ Such perspectives are inherently vulnerable to survivorship bias, and do not examine patients in the context of a hypothetical occult cancer risk influencing treatment choices, which is unique to decision-making in early OG cancer.

Where studies which explored patient preferences prospectively in the context of choosing between definitive surgery and organ-preserving strategies, these have been limited to patients with locally advanced disease, rather than early cancer. Hermus *et al*. interviewed patients regarding the choice between esophagectomy and active surveillance after a complete response to neoadjuvant chemotherapy (NAC) in the SANO study.^19^^,^^20^ Similar to our findings, participants in the active surveillance group prioritized quality of life. However, although these patients also faced a hypothetical risk in the form of occult residual tumor, most of these patients had disease staged as T3 or above, and had already undergone NAC, placing them in a substantially different clinical context from the post-ER population examined in the present study,

In our study, while some patients were strongly in favor of prioritizing quality of life to avoid surgery, others reported that the repeated investigations required for endoscopic surveillance, or cancer-related anxiety this might entail, might sway them in favor of surgery instead. For some participants, surgical resection was perceived as offering greater certainty regarding cancer clearance through reduction of nodal recurrence risk, thereby mitigating the psychological burden associated with ongoing uncertainty under surveillance strategies. This reflects the importance of how individual patients perceive and respond to ongoing uncertainty when considering treatment options. While previous studies have reported that patients who have undergone ER generally regarded the ongoing surveillance as a reassuring part of on-going care, rather than a source of anxiety,^11^ some patients in the present study clearly felt the opposite. The use of the modified CWS in this study highlighted that anxiety levels are variable between patients, and that they may peak in the immediate post-ER period, and then decrease with time. It is unclear whether this reduction reflects adaptation, reassurance from follow-up surveillance, or both. Nonetheless, cancer-related anxiety was present in many participants, even among those with a lower disease burden following ER. This aligns with prior studies showing that anxiety is not necessarily proportional to disease severity or prognostic indicators,^9–11^ and highlights the need for individualized patient-centered care.

The observed reduction in cancer-related anxiety over time also raises the possibility that treatment priorities may evolve throughout the surveillance pathway. However, cancer-related anxiety is likely to be dynamic rather than static, potentially fluctuating around surveillance endoscopies or additional endoscopic resections where these are required. Although this study was not designed to evaluate temporal changes in treatment preferences, future longitudinal studies would help determine how patient priorities change throughout surveillance and whether these changes influence treatment decision-making.

Furthermore, our data demonstrated a significant correlation between higher CWS and heightened patient concern regarding repeated endoscopic surveillance. This may suggest that patients with elevated cancer-related worry or anxiety may be more likely to consider surgical management in the context of clinical equipoise, as surgery may alleviate the psychological burden associated with ongoing endoscopic appointments required under surveillance strategies. However, these findings should be interpreted cautiously given the modest sample size, and further studies are needed to determine whether these associations are reproducible. Whether tools such as the CWS have a role in supporting clinical decision-making remains to be established.

The results of this study may have several implications for clinical practice. First, they highlight that preoperative counseling should focus not only on survival and recurrence statistics but also on the aspects of treatment that patients themselves identified as most important, such as independence, daily functioning, and long-term quality of life. In current practice, post-ER counseling is often delivered within a gastroenterology-led pathway, with surgical input introduced following MDT discussion where indicated, which may influence how treatment options are framed and understood by patients. These findings support the importance of balanced multidisciplinary counseling involving both surgical and endoscopic expertise when discussing management options following ER. Furthermore, clinicians should adopt flexible communication strategies to accommodate patients’ differing tolerance for information detail and involvement in decision-making. It is also important to recognize that while statistical information and risk predictions may be useful to clinicians, such data can often be overwhelming for patients and may, in fact, impede their understanding.

This study’s design allowed for detailed exploration of patient priorities and perceptions, and the inclusion of participants from multiple geographic regions, enhancing generalizability. Data saturation was achieved, supporting the completeness of the themes identified. However, limitations include the hypothetical nature of the conversations had during the interviews. Patients may of course feel differently when presented with the decision in a real-world clinical setting, where clinician recommendations, family discussions, and the opportunity to take time and reflect on the available options may alter priorities and influence treatment decisions. Furthermore, only patients who remained under endoscopic surveillance, and had not undergone an esophagectomy, were included. This was an intentional design choice to minimize the influence of hindsight, survivorship bias, and postoperative experiences on treatment preferences. Consequently, the perspectives of patients who proceeded to surgery were not captured and remain an important area for future research. Furthermore, while patients were recruited from a broad geographic area across multiple centers, some geographical and demographic limitations remain (such as higher than average socioeconomic index compared to the rest of the country) which may limit generalization. In addition, the patients included in this study were a slightly older and more male demographic than the UK surgical population, which may potentially have influenced some opinion, for example in relation to independence, functional recovery, and tolerance of surgical risk.^21^ Whilst socioeconomic data were derived from publicly available national (postcode) government-level data, specific educational or income levels were not captured.

In conclusion, decision-making following ER for early OG cancer involves a complex balance between the hypothetical risk of recurrence against the tangible impact of surgery on quality of life. Long-term quality of life and independence are central to patient priorities, often outweighing numerical estimates of surgical risk. Flexible, patient-centered counseling that addresses both physical and emotional concerns is essential to support informed decision-making and align treatment with individual patient values. These insights may support more nuanced, patient-centered counseling, improve shared decision-making, and ultimately enable treatment strategies that better align with individual patient values and preferences.

## Data Availability

The data that support the findings of this study are available from the corresponding author upon reasonable request.

## Notes

Specific author contributions: Kirsty Cole (Conceptualization, Data curation, Formal analysis, Investigation, Methodology, Project administration, Writing—original draft, Writing—review & editing), Pradeep Bhandari (Conceptualization, Data curation, Methodology, Writing—review & editing), Michael White (Conceptualization, Data curation, Methodology, Writing—review & editing), Tim Underwood (Conceptualization, Methodology, Writing—review & editing), Sheraz Markar (Conceptualization, Methodology, Writing—review & editing), Philip H. Pucher (Conceptualization, Data curation, Formal analysis, Funding acquisition, Investigation, Methodology, Project administration, Supervision, Writing—original draft, Writing—review & editing).

Financial support: The Wessex Cancer Alliance have provided financial support for this study.

## References

[1] Noordzij I C, Curvers W L, Schoon E J. Endoscopic resection for early esophageal carcinoma. J Thorac Dis 2019; 11: S713–22. 10.21037/jtd.2019.03.19 PMC6503291 31080649

[2] Gotink A W, Van De Ven S E M, Ten Kate F J C et al. Individual risk calculator to predict lymph node metastases in patients with submucosal (T1b) esophageal adenocarcinoma: a multicenter cohort study. Endoscopy. 2022; 54: 109–17. 10.1055/a-1399-4989 33626582

[3] NICE . NICE Guidelines [Internet]. 2023; [cited 2025 Feb 2]. Barrett’s oesophagus and stage 1 oesophageal adenocarcinoma: monitoring and management. Available from: https://www.nice.org.uk/guidance/ng231.

[4] Pucher P H, Rahman S A, Bhandari P et al. Prevalence and risk factors for malignant nodal involvement in early esophago-gastric adenocarcinoma. Ann Surg 2024; 281: 363–70. 10.1097/SLA.0000000000006496 PMC11809703 39219545

[5] Luan Nguyen C, Tovmassian D, Isaacs A, Falk G L. Risk of lymph node metastasis in T1 esophageal adenocarcinoma: a meta-analysis. Dis Esophagus 2024;37:doae012. 10.1093/dote/doae012 38391209

[6] Nevo Y, Ferri L. Current management of gastric adenocarcinoma: a narrative review. J Gastrointest Oncol 2023; 14: 1933–48. 10.21037/jgo-22-818 PMC10502549 37720442

[7] Van Der Wilk B J, Spronk I, Noordman B J et al. Preferences for active surveillance or standard oesophagectomy: discrete-choice experiment. Br J Surg 2022; 109: 169–71. 10.1093/bjs/znab358 34750625

[8] Noordman B J, de Bekker-Grob E W, Coene P P L O et al. Patients’ preferences for treatment after neoadjuvant chemoradiotherapy for oesophageal cancer. Br J Surg 2018; 105: 1630–8. 10.1002/bjs.10897 29947418

[9] Rosmolen W D, Nieuwkerk P T, Pouw R E, van Berge Henegouwen M I, Bergman J J G H M, Sprangers M A G. Quality of life and fear of cancer recurrence after endoscopic treatment for early Barrett’s neoplasia: a prospective study. Dis Esophagus 2016;30:1–9. 10.1111/dote.12512 27766707

[10] Rosmolen W D, Boer K R, de Leeuw R J et al. Quality of life and fear of cancer recurrence after endoscopic and surgical treatment for early neoplasia in Barrett’s esophagus. Endoscopy. 2010; 42: 525–31. 10.1055/s-0029-1244222 20539974

[11] Rosmolen W D, Pouw R E, Bergman J J, Sprangers M A G, Nieuwkerk P T. Reasons for fear of cancer recurrence after endoscopic treatment of T1 esophageal adenocarcinoma. A semi-structured interview study. Dis Esophagus 2024; 37:doae067. 10.1093/dote/doae067 PMC11605615 39169835

[12] Tong A, Sainsbury P, Craig J. Consolidated criteria for reporting qualitative research (COREQ): a 32-item checklist for interviews and focus groups. Int J Qual Health Care 2007; 19: 349–57. 10.1093/intqhc/mzm042 17872937

[13] De Bekker-Grob E W, Niers E J, Van Lanschot J J B, Steyerberg E W, Wijnhoven B P L. Patients’ preferences for surgical management of esophageal Cancer: a discrete choice experiment. World J Surg 2015; 39: 2492–9. 10.1007/s00268-015-3148-8 PMC4554743 26170156

[14] Custers J A E, van den Berg S W, van Laarhoven H W M, Bleiker E M A, Gielissen M F M, Prins J B. The Cancer Worry Scale: detecting fear of recurrence in breast cancer survivors. Cancer Nurs 2014;37:E44–E50. 10.1097/NCC.0b013e3182813a17 23448956

[15] Hsieh H F, Shannon S E. Three approaches to qualitative content analysis. Qual Health Res 2005; 15: 1277–88. 10.1177/1049732305276687 16204405

[16] Cohen J . A power primer. Psychol Bull 1992; 112: 155–9. 10.1037//0033-2909.112.1.155 19565683

[17] Bennett A E, O’neill L, Connolly D et al. Perspectives of esophageal cancer survivors on diagnosis, treatment, and recovery. Cancers (Basel) 2021; 13: 1–12. 10.3390/cancers13010100 PMC7796170 33396253

[18] Malmström M, Klefsgård R, Johansson J, Ivarsson B. Patients’ experiences of supportive care from a long-term perspective after oesophageal cancer surgery - a focus group study. Eur J Oncol Nurs 2013; 17: 856–62. 10.1016/j.ejon.2013.05.003 23732012

[19] Hermus M, van der Wilk B J, Chang R T H et al. Patient preferences for active surveillance vs standard surgery after neoadjuvant chemoradiotherapy in oesophageal cancer treatment: the NOSANO-study. Int J Cancer 2023; 152: 1183–90. 10.1002/ijc.34327 PMC10092042 36250325

[20] Noordman B J, Wijnhoven B P L, Lagarde S M et al. Neoadjuvant chemoradiotherapy plus surgery versus active surveillance for oesophageal cancer: a stepped-wedge cluster randomised trial. BMC Cancer 2018; 18: 142. 10.1186/s12885-018-4034-1 PMC5801846 29409469

[21] Pucher P H, Park M H, Cromwell D A et al. Diagnosis and treatment for gastro-oesophageal cancer in England and Wales: analysis of the National Oesophago-Gastric Cancer Audit (NOGCA) database 2012-2020. Br J Surg 2023; 110: 701–9. 10.1093/bjs/znad065 PMC10364547 36972221

